# Youth-Elder Co-Learning Model in Psychiatric Long-Term Care Education: Mixed Methods Evaluation of Communication and Empathy Outcomes

**DOI:** 10.2196/82812

**Published:** 2026-07-08

**Authors:** Ke-Hsin Chueh, Jeng-Wen Chen, Li-Ang Lee, Hsiang-I Hsu, Yen-Ju Lin, Hsiao-Tzu Wang

**Affiliations:** 1 Department of Nursing College of Medicine Fu Jen Catholic University New Taipei City Taiwan; 2 Bachelor Degree Program of Long-Term Care and Health Management College of Medicine Fu Jen Catholic University New Taipei City Taiwan; 3 Center for Medical Education College of Medicine Fu Jen Catholic University New Taipei City Taiwan; 4 Department of Otolaryngology-Head and Neck Surgery Cardinal Tien Hospital and College of Medicine Fu Jen Catholic University New Taipei City Taiwan; 5 Department of Otolaryngology–Head and Neck Surgery National Taiwan University Hospital Taipei Taiwan; 6 Department of Hospital Management Graduate Institute of Business Administration Fu Jen Catholic University New Taipei City Taiwan; 7 School of Medicine, College of Life Science and Medicine National Tsing Hua University Hsinchu Taiwan; 8 Department of Otorhinolaryngology - Head and Neck Surgery Linkou Main Branch Chang Gung Memorial Hospital Taoyuan Taiwan; 9 School of Medicine College of Medicine Chang Gung University Taoyuan Taiwan; 10 MS Program in Transdisciplinary Long Term Care College of Medicine Fu Jen Catholic University New Taipei City Taiwan; 11 Department of Nursing Lo-Sheng Hospital Ministry of Health and Welfare New Taipei City Taiwan; 12 Department of Nursing Bali Psychiatric Center Ministry of Health and Welfare New Taipei City Taiwan; 13 School of Nursing, College of Medicine National Taiwan University Taipei Taiwan

**Keywords:** community psychiatric long-term care, LTC, youth-elder co-learning, professional knowledge, communication competence, empathy, long-term care

## Abstract

**Background:**

Taiwan is projected to become a “super-aged” society by 2025, leading to an increasing demand for community psychiatric long-term care (LTC). This demographic shift necessitates frontline professionals equipped with specialized communication skills and deep empathy. However, traditional didactic teaching often fails to adequately prepare students for the complex emotional and practical challenges of real-world psychiatric caregiving.

**Objective:**

This study aimed to evaluate the effectiveness of the innovative youth-elder co-learning instructional model, which uniquely integrates micro-movie discussions and an intergenerational empathy board game, on adult learners’ professional knowledge, communication competence, empathic development, and overall learning satisfaction.

**Methods:**

A mixed methods, single-group, pre-post design was used. The educational intervention was implemented within an 18-week elective community psychiatric LTC course. Participants included 38 adult learners and continuing education students (aged 19-64 years). Notably, the majority of the cohort (n=29, 76.3%) had no prior practical experience in LTC. Quantitative data were collected using self-assessed and peer-evaluated scales for professional knowledge, communication competence, and empathy at pre-, mid-, and post-course time points, alongside an end-of-semester course student feedback survey. Qualitative data were systematically gathered through structured reflective journals and analyzed using a rigorous 6-phase thematic analysis framework.

**Results:**

Students reported high course satisfaction rates, ranging from 92.4% to 95.3%. Quantitative analysis revealed a notable divergence: there were significant improvements in peer-evaluated outcomes (*P*<.001) and self-assessed communication competence (*P*=.004), but there was more conservative, statistically nonsignificant growth in self-assessed scores for professional knowledge (*P*=.14) and empathy (*P*=.09). This discrepancy likely reflects adult learners’ heightened awareness of professional complexity and self-reflective humility. Furthermore, the qualitative thematic analysis uncovered the following three narrative shifts: (1) the dismantling of generational stereotypes through authentic, face-to-face interaction with real older adults; (2) an empathic awakening regarding the often-invisible burden of family caregivers, catalyzed by the micro-movies; and (3) the successful translation of theoretical nonviolent communication concepts into real-time clinical problem-solving during board game role-plays.

**Conclusions:**

The youth-elder co-learning model shows promise as an innovative, experiential pedagogical approach. By bridging theoretical frameworks with authentic intergenerational contact, the intervention supported students in translating general empathic concepts into actionable communication competencies. However, given the exploratory nature of this study and the absence of a control group, the quantitative findings must be interpreted cautiously. Future research using randomized controlled trial designs across multiple institutions is warranted to establish definitive causal impacts.

## Introduction

The global aging population, with an increasing proportion of individuals aged 65 years and older, presents significant implications for health care education and societal structures [1]. Taiwan is on track to become a “super-aged society” by 2025, where over 20% of its population will be aged 65 years and above, intensifying the need for effective care and mental health services for older adults [2,3]. Mental disorders contribute substantially to the global burden of disease, with disability-adjusted life years and years lived with disability showing notable increases [4]. In Taiwan, approximately 187,000 individuals hold chronic mental illness cards, and this population experiences a shorter median life expectancy compared to the general population [5,6].

The chronic, recurrent, and functionally debilitating nature of mental illness, along with its associated economic burden, often places significant strain on caregivers [7]. Critical health care concerns include reducing relapse rates, facilitating patient community reintegration, and alleviating caregiver burden [8-10]. Despite the high demand for community psychiatric services, there is a widespread lack of public understanding regarding mental health issues, contributing to stigma and discrimination in various aspects of life, including health care, housing, and employment [11]. Stigma can worsen patient adaptation, self-esteem, and depression [12,13]. In health care, mental illness is often viewed as the primary issue, overshadowing other physical health concerns, leading to discriminatory attitudes and unequal treatment [14-16]. Caregivers’ stigmatizing attitudes can further reduce empathy and support, while a lack of professional knowledge may compromise care quality [17,18]. Therefore, universities must equip students with accurate mental health care knowledge and empathetic attitudes to counter stigma [19]. Inclusive education principles should be adopted in promoting community psychiatric long-term care (LTC) education to eliminate discrimination and prejudice, ensuring equal access to learning and care opportunities [20].

The educational effectiveness of the youth-elder co-learning model encompasses 3 core mechanisms. First, intergenerational dialogue enables young and older participants to exchange experiences and perspectives on an equal footing, thereby fostering mutual understanding and empathy while challenging generational stereotypes [21]. Second, based on the theory of social constructivism, knowledge is co-constructed through authentic contexts and social interaction; by engaging in concrete tasks and collaborative activities, students can internalize abstract concepts into practical competencies [22]. Third, reciprocal learning emphasizes that both young and older individuals serve as learners and knowledge contributors. Through skill exchange and joint participation, participants enhance mutual understanding and respect, which can help mitigate generational stereotypes and encourage positive adjustments in professional attitudes [23].

In summary, the “youth-elder co-learning” teaching method facilitates young people’s understanding of the life circumstances and challenges faced by older adults, thereby enhancing their empathy and sense of social responsibility [3,24]. Furthermore, it has a significant positive impact on young people’s attitudes toward older adults, their comfort in interacting with them, and their interest in pursuing careers related to older adult care [23]. Within the youth-elder co-learning model, “micro-movies” and “empathy board games” serve as key interactive platforms to promote mutual understanding and empathy in community-based mental health care [24-28]. Micro-movies offer a narrative structure that enhances character and plot connections, accelerates story progression, and effectively uses classroom time [29]. They are effective tools for public communication and help learners understand the world, with storylines often based on real-life events and incorporating conflicts to leave a lasting impression [30,31]. Empathy board games combine education and communication in a playful format that is applicable across a wide age range [32-35]. They provide experiences that deepen understanding from different perspectives, identify alternative solutions, and encourage critical reflection and interpersonal communication [36-39]. These games enhance understanding, learning, creativity [40,41], active participation [42], behavioral change [43], critical thinking [44], memory [39], and self-esteem [33]. The game dynamics create a positive atmosphere, changing learners’ attitudes toward the content and encouraging empathy for characters, extending to real-life situations [45,46]. By successfully integrating local knowledge and culture, as demonstrated in Taiwan, board games can foster intergenerational communication and understanding [47]. In LTC environments, they can enhance caregiver-patient interaction and understanding, thereby improving physical and mental health [26,48]. Furthermore, cultivating and accurately evaluating empathy remains a cornerstone of professional health care education [49], which can be effectively measured using culturally validated instruments such as the Chinese version of the Jefferson Scale of Empathy [50].

Therefore, the development of an effective educational platform for community psychiatric LTC, incorporating youth-elder co-learning, micro-movies, and empathy board games, represents a valuable avenue. Traditional lecturing often falls short in preparing students for the subtle emotional demands of psychiatric care. This gap is particularly pronounced among nontraditional college students and adult learners in continuing education programs. Although these students often possess diverse life and professional experiences, they frequently lack specialized exposure to community psychiatric care environments. Selecting this population provides an opportunity to evaluate how interactive instructional models can support the connection between existing general life competencies and specialized clinical empathy. This approach is designed to support in-service students balancing work and study by facilitating interactive learning within restricted timeframes, managing knowledge depth, addressing mental illness stigma, and fostering self-efficacy. The youth-elder co-learning model guides students to interact and communicate with patients and caregivers, encouraging the development of professional knowledge, competence, and empathy.

## Methods

### Study Design

This study used a mixed methods, single-group pre-post design to evaluate the educational effectiveness of the youth-elder co-learning instructional model within a university setting. The reporting of this study adheres to the TREND (Transparent Reporting of Evaluations with Nonrandomized Designs) guidelines for the quantitative intervention evaluation and the SRQR (Standards for Reporting Qualitative Research) guidelines for the qualitative components.

### Setting

The research was conducted within an 18-week elective community psychiatric LTC course at a private university. The course is a required component of the Continuing Education Degree Program in LTC and Health Management and is also available as an elective to students from other departments. The setting provided an authentic academic environment, deliberately structured to balance foundational theory with intensive practical application.

The study and data collection spanned the entire semester, with specific intervention periods occurring during the middle of the course (weeks 6-8) and late in the course (weeks 14-15). Data collection was strategically aligned with the curriculum. To provide a clear overview of the study’s procedural sequence, the 18-week curriculum was systematically mapped. Table 1 illustrates the chronological integration of theoretical lectures and the specific timing of the youth-elder co-learning practical interventions, as well as the timeline for pre-, mid-, and post-course evaluations.

**Table 1:** Timeline of the youth-elder co-learning educational intervention process and data collection.

Time point	Educational content and intervention	Data collection and evaluation
Week 1	Course orientation, informed consent, and introduction to community psychiatric LTC^a^	Precourse evaluation: self-assessed knowledge, communication, and empathy scales
Weeks 2-5	Theoretical scaffolding: lectures on psychiatric symptom assessment, caregiver burden, and nonviolent communication	—^b^
Week 6	Youth-elder co-learning intervention phase 1: micro-movie A^c^ discussion (with older adults)	—
Week 7	Holiday	—
Week 8	Youth-elder co-learning intervention phase 1: empathy board game practicum (with older adults)	—
Week 9	Midterm review and reflection	Midcourse evaluation: self-assessed scales and peer evaluation; qualitative: reflective journal
Weeks 10-13	Theoretical scaffolding: lectures on depression, suicide prevention, and substance abuse	—
Week 14	Youth-elder co-learning intervention phase 2: micro-movie B^d^ discussion (with older adults)	—
Week 15	Youth-elder co-learning intervention phase 2: empathy board game practicum (with older adults)	—
Week 16	Course wrap-up and final reflection	Postcourse evaluation: self-assessed scales; qualitative: reflective journal
Week 17-18	Flexible self-directed learning weeks	Final evaluation: peer evaluation and Course Student Feedback Survey

^a^LTC: long-term care.

^b^Not applicable.

^c^Micro-movie A: the patient.

^d^Micro-movie B: unintentional mistake.

### Participants and Study Size

The target population for this study was intentionally selected from adult learners and in-service students enrolled in the community psychiatric LTC course. Participants were recruited using convenience sampling. The eligibility criteria for inclusion were (1) official enrollment in the course during the study semester and (2) provision of voluntary informed consent to participate in the study. Students who did not consent were excluded from the data collection but still participated in the regular course activities without penalty. Regarding the study size, it was intrinsically determined by the total enrollment capacity of the elective course for that semester. A formal a priori statistical power analysis was not conducted because the maximum possible sample size was fixed by institutional course limits.

Consequently, an initial cohort of 38 students who met the eligibility criteria formed our sample. Although the sample size was determined by institutional enrollment limits for the elective course, a post hoc power analysis conducted using G*Power software (version 3.1; Heinrich Heine University Düsseldorf) indicated that, with N=38 and α=.05, the study achieved a power (1–*β*) of 0.92 for detecting medium to large effect sizes. Given the exploratory nature of this intervention, this level of power suggests that the sample size was sufficient for a formative evaluation of the effectiveness of the youth-elder co-learning model.

**Figure 1:**
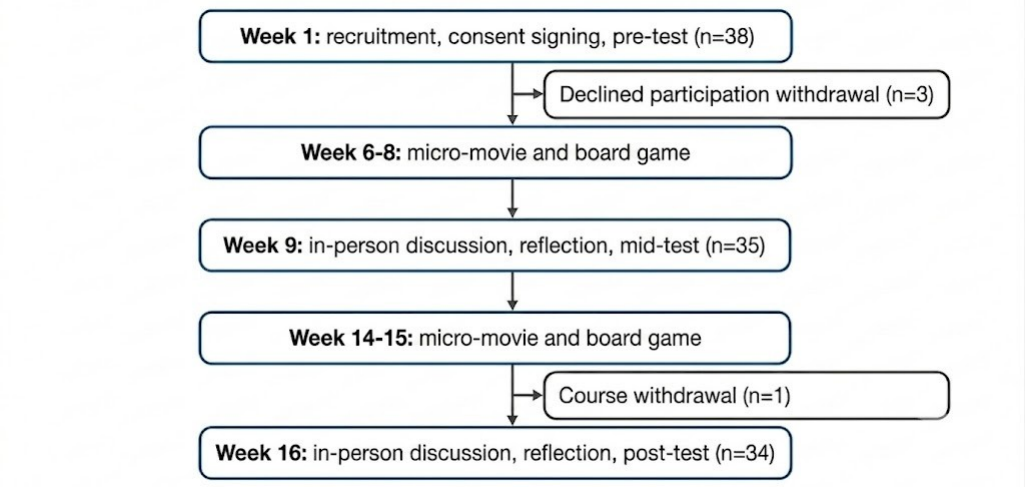
Research process flow.

Demographically, these participants were primarily second-year students spanning a wide age range (19-64 years). Crucially, the majority (29/38, 76.3%) reported having no prior practical experience in LTC or mental health care, making this cohort an ideal target population for evaluating an empathy- and communication-focused intervention. This student population represents a distinct cohort of adult learners and continuing education students who juggle full-time professional responsibilities with academic pursuits. While they bring mature cognitive frameworks to the classroom, their lack of clinical psychiatric familiarity often leads to implicit generational stereotypes or low caregiving confidence. Consequently, investigating this specific college student population allows for a robust assessment of how a targeted educational intervention can shape the professional competence of future frontline LTC providers. Regarding sampling sufficiency for the qualitative component, the inclusion of all 38 enrolled students ensured a comprehensive capture of diverse perspectives within this specific educational context. Data saturation was considered achieved as the analysis of the 38 reflective journals reached a point where no new themes or significant insights related to the youth-elder co-learning model emerged, and the identified patterns—such as the dismantling of generational stereotypes and empathic awakening—became consistently recurrent across the dataset. The research process flow is provided in Figure 1.

### The Youth-Elder Co-Learning Intervention

The youth-elder co-learning model was strictly guided by social constructivism. To put this theory into practice, the intervention was systematically organized into two primary modalities: micro-movie discussions and empathy board game practicums. Regarding the frequency and duration of the program, older adults were invited to the classroom during weeks 6-8 and weeks 14-15. In total, students and older adults met face-to-face for 4 dedicated intergenerational sessions, each lasting exactly 120 minutes. During the micro-movie discussions modality, students and older adults coviewed narrative micro-movies depicting authentic psychiatric LTC dilemmas, which were immediately followed by facilitated discussions to analyze caregivers’ motives and identify potential support strategies.

In terms of the implementation procedures of the empathy board game, the practicums followed a strict 4-step sequential protocol to ensure high intervention fidelity and replicability. First, during the group setup phase, mixed groups comprising 4-5 students and 1-2 older adults were formed. Second, the scenario presentation phase required participants to draw cards that depicted authentic community psychiatric LTC challenges. Third, during the role-play and mediated interaction phase, participants used designated “emotion cards” to articulate hidden feelings, which actively encouraged students to practice nonviolent communication. Finally, the entire session concluded with a structured debriefing phase, which consisted of a 60-minute gameplay period followed by a 60-minute debriefing centered on emotional dynamics and practical clinical application.

### Assessments and Data Sources

To mitigate measurement bias, data were sourced through multiple methods, incorporating 5 distinct quantitative measurement instruments. Specifically, the quantitative data sources comprised 4 self-assessed scales and one peer-assessed instrument. First, the self-assessed knowledge of community mental health LTC was measured using a 20-item scale developed by the research team, which demonstrated a content validity index (CVI) of 1.00 and a Kuder-Richardson 20 reliability coefficient of 0.64. Second, self-assessed communication competency was evaluated using a 0-10 scale focused on nonviolent and therapeutic communication skills, yielding a CVI of 1.00 and a Cronbach α of 0.96. Third, self-assessed empathy was captured via a modified 20-item Jefferson Scale of Empathy for Health Professions, which exhibited a CVI of 1.00 and a Cronbach α of 0.87. Fourth, to objectively assess the practical application of these competencies, a peer-assessed knowledge, communication, and empathy of community mental health scale was administered using a 5-point Likert rating system, demonstrating a CVI of 1.00 and a Cronbach α of 0.94. Finally, a self-assessed end-of-course student feedback survey used a 1-5 scale to measure perceived pedagogical benefits, students’ preferences, and overall satisfaction with the teaching approach, showing a CVI of 1.00 and a Cronbach α of 0.92. To provide full transparency, the complete items for these designated measurement tools are detailed extensively in Multimedia Appendix 1.

Complementing these quantitative metrics, qualitative data were collected from reflective journals submitted voluntarily by participants at weeks 9 and 16. The original purpose of these journals was to serve as a structured formative assessment to capture students’ internal cognitive shifts, emotional processing of caregiving burden, and real-time reflections during the intergenerational co-learning process. To ensure the depth of data, students were instructed to complete these journals independently in a private setting and were explicitly guided to move beyond superficial behavioral summaries, focusing instead on profound self-reflection regarding their interactive dynamics with the older adults. To systematically scaffold their responses and elicit deep reflection, students were provided with five specific semistructured guiding questions. First, students reflected on their initial impressions and subsequent changes in perspective regarding older adults with chronic mental illness after the co-learning sessions. Second, they evaluated the specific communication challenges encountered during the board game practicums and the strategies used to resolve them. Third, the questions prompted students to analyze how the narrative micro-movies altered their subjective understanding of caregiving burden and family dynamics in psychiatric LTC. Fourth, students examined the ways in which the intergenerational dialogue stimulated their evolving professional empathy and clinical values. Finally, they articulated their comprehensive personal insights and future caregiving confidence regarding frontline community LTC.

### Mixed Methods Data Integration

To ensure a robust and comprehensive evaluation of the youth-elder co-learning model, this study adopted a convergent parallel mixed methods design, wherein quantitative and qualitative data were collected concurrently during the 18-week intervention but were analyzed independently to maintain analytical integrity. The programmatic integration and triangulation of these 2 data streams were formally executed during the final interpretation and discussion phase using a joint-display triangulation protocol. Specifically, the quantitative findings from the self-assessed and peer-evaluated metrics served to establish the statistical magnitude, significance, and longitudinal trajectories of students’ development in professional knowledge, communication competence, and empathy. Concurrently, the qualitative insights extracted from the thematic analysis of reflective journals were used to yield a deeper contextual understanding, effectively explaining the underlying mechanisms of the quantitative shifts by illustrating how and why students processed caregiving burdens and dismantled implicit generational stereotypes.

Through this convergence, the qualitative narratives directly complemented the statistical outcomes, providing concrete behavioral examples of empathic awakening that corresponded with the quantitative score increases. Furthermore, any convergent discrepancies—most notably the distinct variance observed between subjective self-assessments and objective peer evaluations—were systematically cross-examined and juxtaposed within the discussion. This integrative approach allowed the research team to mitigate potential measurement biases, maximize data validation, and construct a highly nuanced, multidimensional assessment of how the interactive instructional intervention shapes the professional competence of future frontline LTC providers.

### Data Analysis and Quality Control

Regarding the quantitative analysis, all statistical data processing was performed using IBM SPSS Statistics for Windows (version 25.0). Descriptive statistics were used to summarize the baseline profiles of the participants. For inferential statistics, nonparametric tests were deliberately chosen due to the relatively small sample size and nonnormal data distribution. Specifically, the Wilcoxon signed-rank test was used to evaluate longitudinal changes across the 3 designated time points, with the threshold for statistical significance predefined at an ɑ level of *P*<.05.

For the qualitative analysis, the qualitative component of this study was grounded in the interpretive paradigm, specifically using social constructivism as the underlying philosophical framework. This paradigm posits that knowledge is not a static reality to be discovered but is actively coconstructed through social interactions and the subjective meanings individuals assign to their experiences. By adopting this stance, the research aimed to explore how students meaningfully reconstructed their understanding of psychiatric care and empathy through the intergenerational dialogue with older adults. To operationalize this framework, the research used inductive thematic analysis, strictly adhering to the 6-phase framework outlined by Braun and Clarke [51]. This specific analytic approach was chosen for its theoretical flexibility and its suitability for an inductive, exploratory study. Given the novel nature of the youth-elder co-learning model, this method allowed patterns and themes to emerge directly from the students’ lived experiences—unconstrained by preexisting theoretical frameworks—ensuring that the findings authentically reflected the nuances of intergenerational interaction.

The qualitative data processing followed a rigorous protocol. First, reflective journals were exported from the university’s online learning platform and stored in secure, password-protected digital folders. During the data management phase, each journal was assigned a unique ID (eg, M1-M38) to ensure participant anonymity. Regarding the specific coding personnel and analytical software used, the systematic thematic coding process was executed manually and independently by 2 primary researchers from the research team who possess comprehensive expertise in qualitative nursing methodologies and health care education. Data entry, tracking, and open coding were managed systematically using a standardized digital matrix within Microsoft Excel. This manual spreadsheet approach facilitated close, iterative engagement with the raw texts, wherein the 2 coders mapped out meaning units—defined as specific sentences or paragraphs conveying a distinct learning experience—and directly paired them with open codes within the spreadsheet. Moving systematically from coding to generating, reviewing, and explicitly defining themes, any discrepancies in interpretation were resolved through iterative discussion between the researchers to ensure analytical rigor and trustworthiness.

In terms of quality control and researcher reflexivity, the research team—comprised of experts in nursing education and LTC management—acknowledged that their professional backgrounds and positive expectations regarding the youth-elder co-learning model could potentially influence the interpretation of the students’ reflective journals. To ensure rigor, maintain high standards of trustworthiness, mitigate such biases, and alleviate power dynamics, the primary course instructor, who held a direct power relationship with the students, was strictly excluded from both the research implementation and analysis phases. Researcher triangulation was achieved through independent coding by 2 researchers, followed by consensus-building sessions to resolve any interpretive discrepancies. Throughout the thematic analysis, the researchers maintained a reflexive stance by documenting their internal reactions to the data and maintaining a detailed audit trail that documented all analytical decisions, coding revisions, and the evolution of theme definitions. Although member checking was not feasible due to the anonymous nature of the submissions, the use of peer debriefing with a senior psychiatric nursing expert further validated that the identified themes were representative and grounded in the clinical educational context. Finally, to further enhance overall quality control and data integrity, all data collection was highly standardized using electronic capture methods (eg, Google Forms).

### Ethical Considerations

To ensure the protection of human participants, this study was conducted in strict accordance with the Declaration of Helsinki and ethical guidelines. The research protocol underwent full review and was formally approved by the Fu Jen Catholic University Institutional Review Board (C112216). Before any data collection, written informed consent was obtained from all participants. During the recruitment phase in week 1, students were thoroughly briefed on the study’s objectives, procedures, and their right to withdraw at any time without penalty. It was explicitly emphasized that participation was entirely voluntary and would not positively or negatively affect their course grades or academic standing. To safeguard privacy and confidentiality, all collected data were strictly deidentified. Quantitative assessments and qualitative reflective journals submitted via the online learning platform were stripped of personal identifiers and assigned unique alphanumeric codes (eg, “student 01”). Furthermore, the primary course instructor was intentionally blinded to the students’ consent status and raw data throughout the semester to mitigate any potential power dynamics. Only independent research team members had access to the securely stored, anonymized dataset. Finally, regarding participant compensation, no financial incentives, gifts, or extra academic credits were provided to the students for their participation in this study.

## Results

### Quantitative Research Results

The study sample consisted predominantly of female participants (30/38, 78.9%). The average age was 34.47 (SD 15.83) years, ranging from 19 to 64 years, with the 21-30-year age group being the largest (21/38, 54.6%). Most students (29/38, 76.3%) reported no prior experience in LTC, indicating a lack of practical experience in the field (Table 2).

**Table 2:** Demographic characteristics of participants (N=38).

Characteristic			Values
**Sex, n (%)**			
	Male	8 (21.1)	
	Female	30 (78.9)	
**Age (years)**			
	Mean (SD)	34.47 (15.83)	
	Range	19-64	
**Age groups (years), n (%)**			
	19-20	2 (5.3)	
	21-30	21 (54.6)	
	31-40	2 (5.3)	
	41-50	2 (5.3)	
	51-60	8 (20.8)	
	>60	3 (7.8)	
**LTC^a^** **experience (years)**			
	Mean (SD)	0.71 (1.73)	
	Range	0-6	
**LTC experience groups (years)**			
	0	29 (76.3)	
	<1	3 (7.8)	
	1 to <2	2 (5.3)	
	5	2 (5.3)	
	6	2 (5.3)	

^a^LTC: long-term care.

The “youth-elder co-learning” teaching model showed significant improvement only in students’ self-assessment of communication skills (*z*=–2.88; *P*<.004 from mid to postcourset and *z*=–1.98; *P*=.048 from pre- to postcourse). However, there was no statistically significant improvement in self-assessments of professional knowledge or empathy. In contrast, peer evaluations demonstrated effective progress in all areas: professional knowledge (*z*=–4.93; *P*<.001), communication skills (*z*=–4.94; *P*<.001), and empathy (*z*=–4.55; *P*<.001) (Table 3).

**Table 3:** Differences in knowledge, competency, and empathy pre- and postintervention (N=38).

Outcome measure			Scores, mean (SD)	Statistical comparison	*z*		*P* value^a^
Knowledge							
	Precourse (T0; n=38)	11.16 (3.15)		Pre-mid	–0.07	.95	
	Midcourse (T1; n=35)	11.29 (3.66)		Pre-post	–1.89	.06	
	Postcourse (T2; n=34)	12.09 (4.05)		Mid-post	–1.49	.14	
Knowledge (peer)							
	Midcourse (T1; n=35)	2.22 (0.68)		Mid-post	–4.93	<.001	
	Postcourse (T2; n=35)	3.22 (0.66)		—^b^	—	—	
Communication							
	Precourse (T0; n=38)	6.45 (2.24)		Pre-mid	–0.01	.99	
	Midcourse (T1; n=35)	6.54 (2.23)		Pre-post	–1.98	.048	
	Postcourse (T2; n=34)	7.53 (1.67)		Mid-post	–2.88	.004	
Communication (peer)							
	Midcourse (T1; n=35)	2.39 (0.65)		Mid-post	–4.94	<.001	
	Postcourse (T2; n=35)	3.31 (0.63)		—	—	—	
Empathy							
	Precourse (T0; n=38)	84.97 (18.88)		Pre-mid	–0.95	.34	
	Midcourse (T1; n=35)	83.06 (17.73)		Pre-post	–0.52	.61	
	Postcourse (T2; n=34)	89.03 (15.50)		Mid-post	–1.67	.09	
Empathy (peer)							
	Midcourse (T1; n=35)	2.45 (0.80)		Mid-post	–4.55	<.001	
	Post course (T2; n=35)	3.33 (0.72)		—	—	—	

^a^*P* values were calculated using the Wilcoxon signed-rank test.

^b^Not applicable.

Overall, student satisfaction with the teaching activities ranged from 92.4% to 95.3%, indicating high acceptance. The micro-movie course and the “youth-elder co-learning” program had the highest satisfaction (95.3%), while the board game course achieved 92.9% satisfaction. Students also reported increased confidence in their ability to work in community mental health LTC after course completion (92.4% satisfaction; Table 4).

**Table 4:** End-of-course student feedback (N=34).

Item	Scores, mean (SD)	Standardized scores^a^ (%)	Ranking
Overall satisfaction with the course arrangement this semester?	4.68 (0.59)	93.5	3
Satisfaction with the “micro-movie” course this semester?	4.76 (0.50)	95.3	1
Satisfaction with the “board game” course this semester?	4.65 (0.65)	92.9	4
Did the “youth-elder co-learning” program improve your satisfaction with community mental health LTC^b^?	4.76 (0.50)	95.3	1
After completing the course, do you feel more confident about working in community mental health LTC^b^?	4.62 (0.65)	92.4	5

^a^Standardized score = (original score / maximum score) × 100%.

^b^LTC: long-term care.

### Qualitative Findings

The thematic analysis of students’ voluntary reflective journals revealed a notable shift in their cognitive and emotional understanding of psychiatric LTC. Rather than viewing older adults solely through a lens of clinical detachment, students described a dynamic process of developing professional awareness. The qualitative data converged into three interconnected narratives: dismantling generational stereotypes, experiencing a heightened empathic awareness regarding caregiver burden, and translating theoretical communication models into practical clinical skills.

### Dismantling Generational Stereotypes Through Authentic Interaction

Initially, some adult learners admitted to holding subtle preconceived notions about older adults in psychiatric settings, often anticipating them to be rigid, defensive, or difficult to engage. However, the direct, face-to-face interactions facilitated by the youth-elder co-learning empathy board game actively disrupted these assumptions. Students noted that the structured yet collaborative environment allowed them to perceive the older adults as multifaceted individuals with rich life histories, rather than merely as care recipients. As one student vividly reflected on this intergenerational bridge, “It was quite special. Their thoughts are actually just as clear as ours. Even though they are much older, they are still learning... interacting with them made me realize they often treat us like their own grandchildren, filled with warmth and love” (M6). This authentic contact forced learners to confront their internal biases, replacing initial apprehension with renewed openness and interpersonal warmth.

### Empathic Awakening and the Recognition of Caregiver Burden

The micro-movie discussions served as a powerful emotional catalyst within the curriculum. By analyzing highly realistic portrayals of psychiatric caregiving dilemmas, students moved beyond superficial “perspective-taking” to experience deep emotional resonance. The visual narratives effectively translated abstract textbook concepts into tangible human struggles, shedding light on the immense, often invisible, psychological pressure borne by family caregivers. A recurring sentiment among the participants was the sudden realization of the systemic and emotional complexities inherent in LTC. One participant powerfully noted, “Caregivers are the ‘second patients’—I truly understand that now. I used to think ‘empathy’ was just imagination, but especially after watching the micro-movie, I seemingly truly ‘saw’ the hardships and struggles of caregivers, and can better appreciate what it means to ‘understand another’s powerlessness and choices’” (M36). This affective shift supported the expansion of their theoretical empathy into an actionable intent to provide holistic support.

### Translating Theory Into Practice via Nonviolent Communication

Perhaps a notable pedagogical outcome extracted from the journals was the students’ ability to translate abstract communication theories into actionable, real-time skills. During the situational role-play segments of the board game, students frequently encountered simulated, high-tension communication breakdowns. By using the specific game mechanics (such as cards representing emotions and moods), they practiced articulating underlying needs and actively listening, directly applying nonviolent communication techniques. Students reported that these moments of simulated friction provided valuable learning opportunities, encouraging them to adapt. “After actually simulating the situations of different roles in the game, I realized that everyone carries different pressures and emotions in their hearts... this experience taught me to listen more and be more inclusive in communication, reminding myself to maintain respect and care no matter who I face,” expressed another student (M3). Consequently, this experiential learning process supported their confidence in managing complex clinical interactions without resorting to authoritative or dismissive behaviors.

Overall, the qualitative data indicate that the course supported the integration of theoretical knowledge while enhancing empathy, cognitive flexibility, tolerance, care, and respect within complex social and caregiving situations. Throughout the youth-elder co-learning intervention, students demonstrated a more nuanced understanding of vulnerable groups. They reported improvements in communication competencies—specifically in active listening, emotional expression, and conflict resolution—alongside increased empathy, emotional awareness, and self-management. These learning outcomes suggest the potential value of the immersive, interactive teaching methods used in the curriculum. By creating genuine emotional connections through micro-movies and intergenerational board games, the pedagogical approach guided students from cognitive knowledge acquisition to a heightened level of affective understanding and empathy, positively impacting both their future professional practice and their daily interpersonal relationships.

### Convergence and Contextualization of Mixed Methods Findings

To present a holistic evaluation of the youth-elder co-learning model, the quantitative statistical trajectories and qualitative narrative insights were actively contextualized together, demonstrating clear cross-methodological convergence and mutuality. Specifically, the significant longitudinal increases observed in the quantitative communication competency and empathy scores were directly illuminated by the students’ concurrent journal reflections. For instance, the statistical improvement in therapeutic communication from pre- to postintervention directly corresponded with qualitative themes describing how the “emotion cards” in the board game prompted students to move past academic theories and actively practice nonviolent communication in real-time intergenerational dialogues. This qualitative context complements the data by offering behavioral evidence corresponding with the observed numeric increase.

Furthermore, the qualitative data deeply informed and clarified complex patterns within the quantitative results, particularly regarding the statistical variance between subjective self-assessments and objective peer evaluations. While the quantitative data showed a temporary plateau in students’ self-assessed empathy scores at midcourse, the qualitative narratives contextualized this anomaly as a notable “empathic awakening.” The journals revealed that as students engaged in deep, face-to-face dialogue with older adults, they developed an intense awareness of the heavy caregiving burden and their own clinical naivety. This newfound professional humility led them to score themselves more critically on self-assessments, even though their objective peer evaluations simultaneously reflected a high level of clinical competence and interpersonal sensitivity. Consequently, by contextualizing these 2 data streams together, the results provide a much more transparent, multidimensional, and authentic representation of how the interactive intervention shapes both the measurable competencies and the internal psychological growth of future frontline LTC providers.

## Discussion

### Principal Findings

The study’s results affirm that the “youth-elder co-learning” model effectively enhanced students’ peer-evaluated professional knowledge, communication skills, and empathy, as well as self-assessed communication competence within the Community Mental Health LTC course. The model successfully fostered interaction and understanding between younger students and older adults (including care recipients and caregivers), thereby deepening learning experiences. These findings strongly support the social constructivist approach, demonstrating that when students and older adults actively coconstruct knowledge through game-based interactions, the translation from theoretical empathy to practical communication competence is significantly enhanced.

### Reflection on Practical Challenges Encountered in Youth-Elder Co-Learning Teaching

A notable discrepancy emerged: students’ self-assessments of professional knowledge and empathy did not show statistically significant improvement, unlike peer evaluations. This suggests that students may hold a more conservative self-perception of their competencies. This phenomenon could be explained by factors such as students’ humble dispositions, self-doubt, or an increased awareness of the complexity of professional roles as the course progressed. As students gained deeper insight into the expectations and challenges of LTC settings, they might have become less confident in self-rating highly. This cautious self-assessment could reflect both modesty and a genuine recognition of the depth and difficulty of the knowledge and empathy in LTC.

Furthermore, the unique demographic characteristics of our sample—primarily adult learners and in-service professionals in a continuing education program—may provide additional context for this cautious self-assessment. Unlike traditional young adult college students, these nontraditional students (spanning ages 19-64 years) bring extensive life experience to the classroom. This maturity may lead to a deeper, more realistic appreciation of the complexities involved in psychiatric LTC, thereby making them more self-reflective, humble, and stringent when evaluating their own professional knowledge and empathic growth.

These findings align with Xu et al [52], who noted that students often underestimate their mastery of complex skills due to uncertainty about performance standards or growing recognition of real-world professional challenges. As formative assessments expose students to authentic tasks, some become more self-critical, revealing that personal values and increased awareness can suppress self-assessment scores, even amid actual progress. Likewise, Collier-Sewell et al [53] found that nursing students’ growing awareness of professional complexity was associated with more critical self-evaluation, a phenomenon that may extend to learners in other applied disciplines. Their findings reinforce the idea that as students deepen their understanding of practice realities, they may become more reserved in assessing their own competence, not due to lack of progress, but due to heightened awareness and reflective caution.

These findings highlight the importance of addressing not only students’ skill and knowledge growth but also shifts in their internal perceptions, cognitive frameworks, and evaluative processes. Teaching assessments should consider how students evolve in their reflective awareness, aligning with previous research that self-assessment can be influenced by personal values, humility, and recognition of real-world complexities [52-54].

### Integration of Micro-Movies and Situational Role-Play in the Community Mental Health LTC Course

The teaching team’s blended approach, fusing micro-movie–based teaching and situational role-playing within the youth-elder co-learning model, effectively integrated emotional engagement with practical application, promoting deeper learning in professional knowledge, communication competence, and empathic development. Micro-movies provided vivid, emotionally rich representations of caregiving scenarios, making abstract issues tangible and allowing students to understand the psychological burdens and moral dilemmas of caregivers [55,56]. Role-playing further enhanced this by enabling active dialogue and empathy-building with peers and older adults, a strategy shown to cultivate professional competencies and deepen affective learning outcomes [57]. This combination encouraged students to reflect on how individual backgrounds, resources, context, and both physical and psychological states influence choices and behaviors. Students reported approaching others with greater openness, reduced judgment, and increased emotional intelligence and interpersonal understanding. These interactive and experiential pedagogical strategies supported both cognitive understanding and the emotional resonance necessary for developing core competencies in LTC education [58,59].

### Comparison With Previous Studies on Empathy and Intergenerational Education

Our findings regarding the enhancement of communication skills and empathy through the youth-elder co-learning model strongly resonate with recent literature on intergenerational education. Consistent with studies by Trujillo-Torres et al [21] and Pillemer et al [23], our qualitative and peer-assessed results demonstrate that structured intergenerational dialogue effectively dismantles generational stereotypes and fosters mutual respect. However, our study extends these foundational concepts by applying them specifically to the high-stress, stigmatized context of community psychiatric LTC, demonstrating that intergenerational co-learning is not only beneficial for general socialization but is also a vital pedagogical tool for specialized clinical communication.

Furthermore, regarding empathy development, our outcomes align closely with recent nursing education research. Cho and Kim [58] and Huang et al [59] emphasized that immersive, simulation-based interventions are highly effective in cultivating empathy among health care students. While many previous studies rely on standardized patients or peer-to-peer role-play, our youth-elder co-learning model integrates real older adults into the simulation via the “empathy board game.” This authentic interaction operationalizes empathy in real-time. It compels students to move beyond passive “perspective-taking” and actively translate theoretical compassion into practical, on-the-spot communication strategies. The detailed reflective feedback and interpersonal interactions reported by our students suggest that combining game-based learning with active intergenerational contact offers valuable pedagogical opportunities to support empathic development beyond standard classroom lectures.

### Limitations

Despite demonstrating meaningful outcomes, this study has several limitations. First, the relatively small sample size and convenience sampling may limit the generalizability of the findings. Second, the self-assessment tools, while carefully developed, were subject to personal bias, influenced by cultural or psychological factors such as modesty or self-criticism. Third, peer assessments, though valuable, may also be affected by interpersonal dynamics within the class. Fourth, the reliance on a single-group pre-post design without a control group at a single university means that we cannot definitively rule out the influence of external confounding factors—such as the natural passage of time or concurrent coursework—on students’ development. Consequently, the findings may strongly reflect the specific institutional culture or the unique demographic makeup of this particular continuing education program. Future research should consider incorporating a control group design and replicating the study across different universities, student populations, and educational settings. Longitudinal studies would also be beneficial to explore the sustained impact of the youth-elder co-learning model on professional competence and empathic communication over time.

### Conclusion

This study suggests that the youth-elder co-learning model—using micro-movies and intergenerational empathy board games—shows promise as an innovative pedagogical approach for students preparing for community psychiatric LTC. By providing authentic, guided interactions with real older adults, the intervention successfully helped adult learners bridge theoretical knowledge with practical communication and empathic skills. The study contributes to the growing evidence supporting experiential and emotionally engaging teaching methods in LTC education, especially within community mental health.

Through this formative evaluation, several key takeaways emerged to guide future educational research. First, the observed discrepancy between students’ self-assessments and objective peer evaluations highlights a critical methodological issue that warrants deeper future exploration. Second, the strategic integration of emotional resonance and cognitive learning can facilitate meaningful improvements in students’ developing communication competence and empathic awareness. Finally, future LTC course designs should increasingly incorporate interactive, reflective, and immersive teaching strategies that simulate real-life caregiving scenarios to cultivate students’ comprehensive clinical abilities.

However, given the exploratory nature of this study and the absence of a control group, these preliminary results must be interpreted cautiously. While peer evaluations and detailed qualitative feedback indicate meaningful learning patterns, the lack of a comparison cohort prevents us from definitively attributing all observed longitudinal improvements solely to the youth-elder co-learning intervention. These findings highlight the potential value of incorporating active intergenerational contact into LTC curricula, but further rigorous research—specifically using randomized controlled trial designs across multiple institutions—is required to establish definitive causal impacts. Ultimately, by combining emotional engagement with cognitive understanding, the youth-elder co-learning approach fosters not only professional knowledge acquisition but also reflexive capacity and interpersonal sensitivity, which are critical for preparing future nursing and health care professionals to meet the complex demands of community psychiatric care.

## Data Availability

The datasets generated and analyzed during this study are not publicly available due to the inclusion of sensitive qualitative reflections from a small participant cohort but are available from the corresponding author on reasonable request.

## Appendix

Multimedia Appendix 1 Study questionnaires and assessment instruments.

## Abbreviations

CVI: content validity index
LTC: long-term care
SRQR: Standards for Reporting Qualitative Research
TREND: Transparent Reporting of Evaluations with Nonrandomized Designs
