# Supplementary material for: Youth-Elder Co-Learning Model in Psychiatric Long-Term Care Education: Mixed Methods Evaluation of Communication and Empathy Outcomes
Source: JMIR Med Educ. 2026 Jul 8;12:e82812. doi: 10.2196/82812 (PMC13392526; doi:10.2196/82812)
Supplement: Multimedia Appendix 1 [file mededu_v12i1e82812_app1.docx]

**Multimedia Appendix 1**

1. **Personal Attributes**
2. Gender: □ (1) Male □ (2) Female □ (3) Non-binary
3. Date of Birth (YYYY/MM): ______ Year ______ Month (Age: ______)
4. Long-term care work experience: □ (1) Yes □ (2) No
5. Work experience (Role/Field): ______; Years of service: _____ years
6. **Self-Assessed Knowledge of Community Mental Health LTC (20-item questionnaire developed by the research team)**

True/False Questions

Instructions: Please judge the accuracy of the following statements. Fill in "O" if the statement is correct and "X" if it is incorrect.

1. In therapeutic communication, it is best to use closed-ended questions to achieve higher communication efficiency.
2. When applying behavior modification techniques, one should avoid prioritizing positive reinforcement (encouragement).
3. Substance abusers are subjects of judicial correction rather than patients in the medical field; medical intervention can only provide supportive therapy for physiological withdrawal.
4. Caregiver burden most commonly occurs at the onset of a caregiving event; therefore, support resources should be strengthened at that time and can be withdrawn once the caregiver has adapted.
5. Experienced caregivers are defined as those who have cared for family members for over three years; because they are familiar with the caregiving tasks, their burden is lower and they are less prone to depression.
6. Guidance or suggestions given by supervisors or workplace seniors may involve harsh language or demands due to personal management styles, but this should not be confused with workplace bullying.
7. Caregiver burden only occurs within kinship (family) relationships.
8. Medical violence mostly occurs under excusable circumstances; if it enters judicial proceedings, the sentence is often reduced based on the situation.
9. When a care recipient feels anxious and uneasy, the likelihood of violent behavior increases.
10. Suicide is sometimes a way for individuals to seek attention; if we continue to provide responses, it may constantly reinforce self-harming or suicidal behavior. The best way is direct confrontation to make the individual face their own problems.
11. Mental illnesses have a high genetic risk. Based on eugenics and humanitarian considerations, care staff should proactively provide birth control information and encourage clients to use contraception or undergo sterilization.
12. Patients with depression generally have pessimistic personalities and do not open up easily.
13. Medical violence refers specifically to physical (bodily) violence.
14. There are usually signs to look for before violent behavior occurs, such as a flushed face, fierce glare, higher pitch or faster pace of voice, and clenched fists.
15. For clients with mental illness, as long as they take medication regularly, there will be no recurrence of the condition.
16. Most clients in day care centers are chronic psychiatric cases who are already very stable; therefore, there should not be too many restrictions, and they should be allowed to arrange their own lives and use day care services flexibly.
17. Workers without professional training who rashly interact with clients with mental illness are highly likely to accidentally provoke them; therefore, contact with psychiatric clients should be minimized as much as possible.
18. Community facilities related to mental disabilities should be located in independent buildings or as far away from residential areas as possible to reduce the impact on the community.
19. When a psychiatric client exhibits obvious disruptive symptoms, they should be isolated as quickly as possible to reduce the impact on others.
20. As a depressed client's condition gradually improves, the risk of suicide also decreases.

#### **Self-Assessed Communication Competency (0-10 scale, developed and modified by research team)**

Instructions for Completion

This scale is designed to assess your perception of your own core competencies in long-term care. There are no right or wrong answers; please respond based on your "authentic experience or thoughts."

"Competency" refers to the underlying foundation of performance, encompassing three dimensions: knowledge, skills, and affect (attitudes/values).

Please circle the degree to which you believe you possess these competencies based on your current feelings.

The level of proficiency ranges from 0 to 10, where 0 means "Not at all " and 10 means " Fully competent."

1. Do you believe that through the current teaching methods, you have acquired the necessary communication competencies?

0 1 2 3 4 5 6 7 8 9 10

Not at all

Fully competent

|  |  |  |  |  |  |  |  |  |  |
| --- | --- | --- | --- | --- | --- | --- | --- | --- | --- |

1. Do you believe that through the current teaching methods, you have acquired the competency of 'Inclusiveness'? (Inclusiveness: The ability to accept and empathize with another person's perspective and needs, even if you internally disagree with them.)

0 1 2 3 4 5 6 7 8 9 10

Not at all

Fully competent

|  |  |  |  |  |  |  |  |  |  |
| --- | --- | --- | --- | --- | --- | --- | --- | --- | --- |

1. Do you believe that through the current teaching methods, you have acquired the competency of 'Respect'? (Respect: Treating groups and cultures different from one's own with an attitude, speech, and behavior based on equality.)

0 1 2 3 4 5 6 7 8 9 10

Not at all

Fully competent

|  |  |  |  |  |  |  |  |  |  |
| --- | --- | --- | --- | --- | --- | --- | --- | --- | --- |

1. Do you believe that through the current teaching methods, you have acquired the competency of 'Caring'? (Caring: Helping others grow and achieve self-actualization, while providing both psychological and practical physical interventions.)

0 1 2 3 4 5 6 7 8 9 10

Fully competent

Not at all

|  |  |  |  |  |  |  |  |  |  |
| --- | --- | --- | --- | --- | --- | --- | --- | --- | --- |

#### **Self-Assessed Empathy (20-item Jefferson Scale of Empathy for health care professionals, modified for LTC students)**

Instructions: Please read each item thoroughly and indicate the extent to which you agree with the statement based on your personal perspective. Each item contains seven options (1-7), defined as follows.

| **item** | **Strongly Disagree** |  |  |  |  |  | **Strongly Agree** |
| --- | --- | --- | --- | --- | --- | --- | --- |
|  | **1** | **2** | **3** | **4** | **5** | **6** | **7** |
| 1. Staff understanding of the feelings of patients and their families does not affect care outcomes. | **□** | **□** | **□** | **□** | **□** | **□** | **□** |
| 2. When staff understand patients' feelings, the patients feel better. | **□** | **□** | **□** | **□** | **□** | **□** | **□** |
| 3. It is difficult for staff to see things from the patient's perspective. | **□** | **□** | **□** | **□** | **□** | **□** | **□** |
| 4. In the patient-provider relationship, understanding a patient's body language is as important as verbal communication. | **□** | **□** | **□** | **□** | **□** | **□** | **□** |
| 5. A staff member's sense of humor contributes to achieving better care outcomes. | **□** | **□** | **□** | **□** | **□** | **□** | **□** |
| 6. Because every individual is different, it is difficult for me to see things from the patient's perspective. | **□** | **□** | **□** | **□** | **□** | **□** | **□** |
| 7. It is not important to pay attention to a patient's emotional reactions during an interview. | **□** | **□** | **□** | **□** | **□** | **□** | **□** |
| 8. Caring about a patient's personal experiences does not affect the outcome of treatment. | **□** | **□** | **□** | **□** | **□** | **□** | **□** |
| 9. When providing care, staff should put themselves in the patient's shoes. | **□** | **□** | **□** | **□** | **□** | **□** | **□** |
| 10. Patients believe that the mere fact of staff understanding their feelings is, in itself, therapeutic. | **□** | **□** | **□** | **□** | **□** | **□** | **□** |
| 11. Patients' illnesses can only be treated medically; staff's care and concern do not help the treatment outcomes. | **□** | **□** | **□** | **□** | **□** | **□** | **□** |
| 12. Inquiring about a patient's life situation does not help in understanding their physical discomfort. | **□** | **□** | **□** | **□** | **□** | **□** | **□** |
| 13. Staff should observe patients' non-verbal behaviors and body language to understand their inner thoughts. | **□** | **□** | **□** | **□** | **□** | **□** | **□** |
| 14. I believe that the expression of emotions has no effect on the treatment of illness. | **□** | **□** | **□** | **□** | **□** | **□** | **□** |
| 15. Empathy is a therapeutic skill; without it, it is difficult to be a successful staff member. | **□** | **□** | **□** | **□** | **□** | **□** | **□** |
| 16. Staff understanding of the emotional states of patients and their families is a crucial component of the clinician-patient relationship. | **□** | **□** | **□** | **□** | **□** | **□** | **□** |
| 17. Staff should attempt to view problems from the patient's perspective to provide better care. | **□** | **□** | **□** | **□** | **□** | **□** | **□** |
| 18. Staff members should not allow themselves to be moved by the emotions between patients and their families. | **□** | **□** | **□** | **□** | **□** | **□** | **□** |
| 19. I do not enjoy reading non-medical literature or appreciating other forms of art. | **□** | **□** | **□** | **□** | **□** | **□** | **□** |
| 20. I believe that empathy is an important factor in patients' treatment. | **□** | **□** | **□** | **□** | **□** | **□** | **□** |

#### **Peer Evaluation of Professional Knowledge, Communication Competencies, and Empathy (0-5 scale, completed by classmates)**

CBME Scale for Community Psychiatric Long-Term Care

Instructions: This scale is designed for clinical instructors to observe and evaluate the learner’s acquisition and demonstration of competencies in community psychiatric long-term care during the "Intergenerational Learning" course. There are no right or wrong answers. Based on the learner's actual participation and performance, please record the "authentic situation" observed. Proficiency is measured from Level 1 to Level 5; please select the level that most accurately reflects the learner’s practical application.

1. Knowledge of Community Psychiatry

| Proficiency Levels | Level 1  Novice | Level 2  Advanced Beginner | Level 3  Competent | Level 4  Proficient | Level 5  Expert |
| --- | --- | --- | --- | --- | --- |
| Based on observation of the learner, please check the demonstrated competencies. | □ Demonstrates a preliminary understanding of how biological, psychological, and social factors influence the patient's condition. | □ Able to perceive how one’s own emotional reactions may potentially affect the patient.  □ Demonstrates a comprehensive understanding of how biological, psychological, and social factors influence the patient's condition. | □ Able to perceive and describe the impact of one’s own emotional reactions on the patient.  □ Able to integrate the patient's background information (biological, psychological, and social) to form a comprehensive assessment. | □ Able to analyze the patient's condition from multiple perspectives (e.g., family, medical history, behavior) and integrate both the patient’s and one's own emotional reactions to achieve a deeper understanding of the issues. | □ Able to explain the patient's condition clearly and comprehensively, providing appropriate recommendations and guidance for the patient. |
| Examples of Learning Content | 1. What is Depression?  2. What are the primary symptoms of depression?  3. Understanding the simple definitions and manifestations of psychiatric symptoms (e.g., anxiety, sleep disturbances). | 1. Able to observe and identify the emotional states of both the patient and oneself. | 1. Recognizes when a patient needs to seek professional help from a psychiatrist or psychologist. | 1. Encourages the patient to express their emotions and provides appropriate responses.  2. Identifies community resources that provide psychological support, such as support groups or counseling centers. | 1. Assists the patient in establishing a stable daily routine, such as regular meal times and sleep schedules.  2. Demonstrates the ability to collaborate with the patient to design a structured daily lifestyle, promoting biological rhythm stability and self-care management. |

1. Communication in Community Psychiatry

| Proficiency Levels | Level 1  Novice | Level 2  Advanced Beginner | Level 3  Competent | Level 4  Proficient | Level 5  Expert |
| --- | --- | --- | --- | --- | --- |
| Based on observation of the learner, please check the demonstrated competencies. | □ Barriers to effective communication. | □ Able to utilize both verbal and non-verbal behaviors to demonstrate respect and establish rapport. | □ Uses active listening and clear language to establish therapeutic relationships with the patient, family, and caregivers.  □ Recognizes how communication barriers impact specific patients. | □ Uses shared decision-making to establish and maintain therapeutic relationships.  □ Modifies strategies to mitigate barriers to effective communication.  □ Establishes therapeutic relationships during complex interactions with patients, families, and caregivers.  □ Recognizes the influence of personal biases and attitudes on communication. | □ Able to guide patients in developing situational awareness and critical self-reflection to foster positive therapeutic relationships. |
| Examples of Learning Content | 1. Proactively greets the patient and introduces oneself (e.g., 'Hello, I am the caregiver assisting you today'). | 1. Explains the process and rationale of activities (e.g., 'We will spend ten minutes walking now; it is very beneficial for your health'). | 1. During clinical scenario discussions, observe whether the content of the learner's discussion aligns with the following criteria: | 1. Collaborates with the patient to discuss solutions (e.g., 'We can complete this in a few steps; do you think that would make it easier?'). | 1. Proactively discusses the patient's needs with family members (e.g., 'His mood has seemed a bit unstable lately; let's work together to find ways to stabilize his emotions'). |

1. Empathy in Community Psychiatry

| Proficiency Levels | Level 1  Novice | Level 2  Advanced Beginner | Level 3  Competent | Level 4  Proficient | Level 5  Expert |
| --- | --- | --- | --- | --- | --- |
| Based on observation of the learner, please check the demonstrated competencies. | □ Able to observe the patient's emotional expressions and non-verbal behaviors. | □ Able to observe and respond to the patient's emotional expressions and non-verbal behaviors.  □ Able to understand the patient's emotions and express recognition and acceptance of those emotions. | □ Able to provide psychological support when encountering a patient's negative emotions in general clinical settings. | □ Able to provide psychological support when facing a patient's negative emotions in complex clinical situations.  □ Facilitates the patient's understanding and regulation of their own emotions, assisting them in developing effective coping strategies. | □ Able to model empathic responses to the patient’s family, thereby enhancing the quality of community-based care. |
| Examples of Learning Content |  | Provides tissues, listens, and stays with the patient when they are crying. | Offers verbal comfort and reassurance to address the patient's concerns. | Guides the patient in expressing and venting emotions (e.g., 'You can tell me, what is bothering you the most?'). | Encourages family members to participate in supportive activities (e.g., 'This group might help you feel less alone; we can try it together'). |

#### **End-Of-Course Student Feedback Survey (developed by research team, measuring perceived benefits, preferences, and overall evaluations of the teaching approach)**

Instructions: Overall Evaluation of the 'Community Psychiatric Long-Term Care' Course (1 = Very Dissatisfied; 5 = Very Satisfied)

| Item | Content | 1 | 2 | 3 | 4 | 5 |
| --- | --- | --- | --- | --- | --- | --- |
| **1.** | Overall satisfaction with the course arrangement this semester? |  |  |  |  |  |
| **2.** | Satisfaction with the "Micro-movie" course this semester? |  |  |  |  |  |
| **3.** | Satisfaction with the "Board Game" course this semester? |  |  |  |  |  |
| **4.** | Did the "youth-elder co-learning" program improve your satisfaction with community mental health LTC? |  |  |  |  |  |
| **5.** | After completing the course, do you feel more confident about working in community mental health LTC? |  |  |  |  |  |
